# Disentangling sensorimotor and cognitive cardioafferent effects: A cardiac-cycle-time study on spatial stimulus-response compatibility

**DOI:** 10.1038/s41598-020-61068-1

**Published:** 2020-03-04

**Authors:** Mauro F. Larra, Johannes B. Finke, Edmund Wascher, Hartmut Schächinger

**Affiliations:** 1 Leibniz Research Centre for Working Environment and Human Factors, 44139 Dortmund, Germany; 2 Division of Clinical Psychophysiology, Institute of Psychobiology, University of Trier, 54290 Trier, Germany

**Keywords:** Neuroscience, Psychology, Cardiology

## Abstract

Cardiac-cycle-time effects are attributed to variations in baroreceptor (BR) activity and have been shown to impinge on subcortical as well as cortical processes. However, cognitive and sensorimotor processes mediating voluntary responses seem to be differentially affected. We sought to disentangle cardiac-cycle-time effects on subcortical and cortical levels as well as sensorimotor and cognitive processes within a spatial stimulus-response-compatibility paradigm employing startling stimuli of different modalities. Air-puffs and white noise-bursts were presented unilaterally during either cardiac systole or diastole while bilateral startle EMG responses were recorded. Modality, laterality and cardiac-cycle-time were randomly varied within-subjects. Cognitive and sensorimotor stimulus-response-compatibility was orthogonally varied between-subjects: Participants (N = 80) responded to the stimuli via left/right button-push made with either the contra- or ipsilateral hand (sensorimotor compatibility) on either the ipsi- or contralateral button (cognitive compatibility). We found that sensorimotor compatible reactions were speeded during systole whereas sensorimotor incompatible ones were prolonged. This effect was independent of cognitive compatibility and restricted to auditory stimuli. Startle was inhibited during systole irrespective of modality or compatibility. Our results demonstrate how differential cardiac-cycle-time effects influence performance in conflict tasks and further suggest that stimulus-response-compatibility paradigms offer a viable method to uncover the complex interactions underlying behavioral BR effects.

## Introduction

The ability to flexibly adapt our behavior according to changes in bodily states is crucial to survival, promoting adequate actions e.g. in times of illness and stress. This ability is mediated by signaling pathways that convey information about peripheral events to the central nervous system, thereby influencing brain activity and potentially psychological state^1^. Beside humoral transmission of messengers essential to the immune and endocrine systems, neural projections originating from organ receptors located in the periphery contribute to altered brain activity. The cardiovascular system is a major source of variations in such viscero-afferent traffic and arterial baroreceptors (BR), mechanoreceptors expressed mainly within the carotid sinus and the aortic arch, are responsible for relaying cardiovascular events to the brain^2^. These stretch-sensitive receptors increase their firing rate in response to tension on the vessel walls and are essential for the homeostatic control of blood pressure and heart rate^3,4^. Afferent fibers project to the nucleus tractus solitarius (NTS) and determine the output of autonomic brain stem centers, thereby regulating cardiac activity and vascular contraction via sympathetic as well as parasympathetic efferents^5,6^. However, baroafferent signals do not remain at the brainstem level but are relayed by the NTS to the reticular formation and higher-order structures such as the thalamus, hypothalamus, amygdala, and cortex^7–9^, providing the neural basis for behavioral effects beyond baroreflex control.

Such an “irradiation” of baroafferent signaling has first been reported by Koch^10^, who observed that invasive BR stimulation would calm down dogs and bring them to lay down and close their eyes. Ever since then, BR activation has been assumed to exert an overall inhibitory influence on the central nervous system. In humans, this is evident in studies employing external suction to increase BR load^11,12^ or making use of natural fluctuations of BR activation during the cardiac cycle. BR fire during the upstroke and plateau of the pulse pressure wave during systole, but are silent during diastole^13–16^. By synchronizing the presentation of brief stimuli with the cardiac cycle it is possible to assess differences in their processing according to such natural fluctuations in cardioafferent traffic. There is a substantial body of evidence demonstrating that BR activation leads to an inhibition of basic sensory and sensorimotor functions including reduced sensory thresholds^17–21^, inhibited spinal and brainstem reflexes^22–27^ and dampened pain perception^28–31^. These findings led to the formulation of the theory of learned hypertension^29,32^, stating that the pain relieving and overall dampening effect of BR activation may function as a reward and thereby trigger behaviors that promote high blood pressure.

Another approach to investigating the behavioral significance of baroafferent signaling is represented by cardiac-cycle-time studies on cortical stimulus processing. It has been shown that cortical potentials evoked by simple auditory and visual stimuli are diminished during the systolic compared to the diastolic phase^33,34^. Moreover, studies assessing cardiac-cycle-time effects on voluntary sensorimotor reactions have found an inhibitory BR effect. As such, responses in simple and choice reaction time tasks are reported to be prolonged during systole in a number of studies^35–38^. However, some studies failed to find an effect of cardiac timing on manual reaction times^39,40^ while others reported differential effects on evaluative and motor components of reaction times^41,42^. More recently, research has begun addressing cardiac-cycle-time effects on the cognitive processes translating stimuli to behavioral outcomes. As opposed to the simple sensorimotor tasks dominating the previous literature, these studies employ cognitively more demanding paradigms typically entailing the processing of conflicts. For instance, it has been found that attentional selection and signal detection accuracy were improved during systole when targets were presented together with or masked by distracting visual stimuli^43,44^. Another study reported improved response inhibition during systole in a stop-signal task^45^. Moreover, in a series of experiments Azevedo *et al*.^46^ showed that the activation of cognitive stereotypes associating black people with threat seems to be potentiated during systole compared to diastole. Finally, memory, attentional and emotional processing of threat signals have been found to be modulated across the cardiac cycle^47–52^.

In light of the above-mentioned findings, the notion of an overall inhibitory BR effect on cortical stimulus processing appears to be overly simplified. A pressing question is whether cognitive and sensorimotor processes are differentially affected and how such differential effects might be related to each other in producing behavorial outcomes. Here, we sought to differentiate BR effects on sensorimotor and cognitive processes mediating voluntary responses in a conflict task. To this end, we employed a spatial stimulus-response-compatibility (SRC) paradigm using auditory and tactile startle probes presented during systole or diastole as imperative stimuli. The concept of SRC refers to the finding that reactions to laterally presented stimuli are faster when response and stimulus locations coincide^53^. This effect is present even when target location is task-irrelevant^54^ and extends to other conceptual similarities between stimuli and responses beyond the spatial domain^55^. Importantly, within *spatial* SRC, compatibility effects may further be distinguished into sensorimotor compatibility, that is correspondence between side of receptor and effector (e.g. “ear-hand-correspondence” for manual reactions to auditory stimuli), and cognitive compatibility denoting conceptual correspondence between stimulus and response locations (e.g. left button presses to stimuli presented on the left). Whereas the former is attributed to direct activation of contralateral motor areas^56–58^, the latter results from an automatic activation of a cognitive stereotype linking congruent stimulus and response codes^53,55,59^. These components may be varied independently from each other by performing SRC tasks with crossed vs. uncrossed hands^57,60–64^. A further level of specificity may be achieved by presenting stimuli in different modalities, which directly affects sensory, but not motor processes or the cognitive representation of spatial attributes. Spatial SRC paradigms with crossed hands thus offer a straightforward method to differentiate between cardiac-cycle-time effects on cortical sensorimotor and cognitive processes mediating voluntary responses. What is more, through employment of startling imperative stimuli and assessment of bilateral startle responses, sensorimotor effects may further be differentiated between subcortical and cortical levels. The startle reaction is a protective brainstem reflex that is activated by intense and abrupt stimuli and induces an immediate eyeblink response^65^. It is typically quantified by measuring electromyographic activity of the orbicularis oculi muscle responsible for lid closure which has repeatedly been shown to be inhibited during the systolic phase of the cardiac cycle^23–25,41^. Therefore, concurrent assessment of startle responses allows for a separation of cardiac-cycle-time effects on brainstem and higher-order CNS structures and at the same time provides a robust control measure for the effectiveness of the manipulation.

We presented 80 participants with air-puffs and white noise-bursts delivered to either the right or left temples or ears, respectively, during cardiac systole or diastole (230 ms vs. 530 ms after peak of R-wave) while bilateral M. orbicularis oculi EMG responses were recorded. Modality, laterality and cardiac-cycle-time were randomly varied between trials. All participants had to respond to the stimuli with left or right button pushes. Cognitive and sensorimotor stimulus-response-compatibility was orthogonally varied between subjects: with respect to the source of stimulation, responses were to be made with either the contra- or ipsilateral hand (sensorimotor compatibility) on either the ipsi- or contralateral button (cognitive compatibility).

## Results

### Startle responses

**Figure 1:**
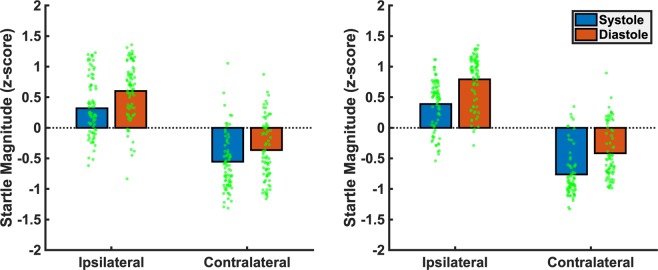
Startle magnitude (z-scored) in systolic (blue) and diastolic (red) trials measured at the eye ipsilateral vs. contralateral to stimulus presentation for auditory (left panel) and tactile (right panel) stimuli.

A mixed-model ANOVA comprising the between-subject factors SM-COMP (sensorimotor compatible vs. incompatible) and COG-COMP (cognitively compatible vs. incompatible) and the within-subjects factors MODALITY (tactile vs. auditory) * PHASE (systole vs. diastole) * SIDE (ipsi- vs. contralateral) conducted on startle response magnitudes revealed significant main effects of PHASE (*F*[1,73] = 36.54, *p* < 0.001, *η*_*p*_^2^ = 0.334) and SIDE (*F*[1,73] = 563.08, *p* < 0.001, *η*_*p*_^2^ = 0.887) along with a significant interaction of MODALITY*SIDE (*F*[1,73] = 6.59, *p* = 0.012, *η*_*p*_^2^ = 0.083). Startle responses were stronger on the side ipsilateral to stimulus presentation compared to the contralateral side, an effect that was more pronounced for tactile (*t*[76] = 18.84, *p* < 0.001, *η*_*p*_^2^ = 0.824) than for auditory stimuli (*t*[76] = 15.54, *p* < 0.001, *η*_*p*_^2^ = 0.758). As indicated by the main effect of PHASE, startle response magnitude was diminished for stimuli presented in the systolic vs. diastolic phase. Importantly, we found no significant interaction of SIDE*PHASE (*F* < 1) or MODALITY*SIDE*PHASE (*F* < 1) indicating that, cardiac-cycle-time effects did not differ between contralateral vs. ipsilateral startle responses, i.e. were not affected by sensorimotor compatibility, see Fig. 1. The interaction of MODALITY*PHASE (*F*[1,73] = 2.93, *p* = 0.098, *η*_*p*_^2^ = 0.037) did not reach significance, nor were there any other significant main or interaction effects (all *F*-values < 1). Also, the effects were similar across experimental groups, as no significant interactions with the between-subjects compatibility factors (SM-COMP, COG-COMP) could be observed (statistics for *F* > 1: PHASE*COG-COMP: *F*[1,73] = 1.41, *p* = 0.238, *η*_*p*_^2^ = 0.019; PHASE*COG-COMP*SM-COMP: *F*[1,73] = 1.27, *p* = 0.263, *η*_*p*_^2^ = 0.017; SIDE*SM-COMP: *F*[1,73] = 1.87, *p* = 0.175, *η*_*p*_^2^ = 0.025; SIDE*SM-COMP*COG-COMP: *F*[1,73] = 2.56, *p* = 0.114, *η*_*p*_^2^ = 0.034).

### Manual reactions

**Figure 2:**
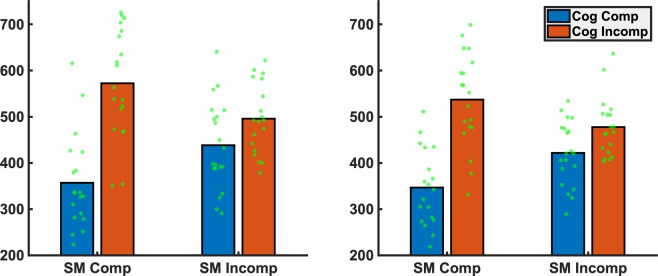
Mean reaction times and individual data points depicted separately as a function of sensorimotor (SM Comp vs. SM Incomp) and cognitive (Cog Comp vs. Cog Incomp) compatibility for auditory (left panel) and tactile (right panel) stimuli.

Manual reaction times were subjected to a mixed-model ANOVA comprising the within-subject factors MODALITY and PHASE as well as the between-subject factors SM-COMP (sensorimotor compatible vs. incompatible) and COG-COMP (cognitively compatible vs. incompatible). We found significant main effects of MODALITY (*F*[1,73] = 11.87, *p* = 0.001, *η*_*p*_^2^ = 0.132), COG-COMP (*F*[1,73] = 43.6, *p* < 0.001, *η*_*p*_^2^ = 0.406) and a significant interaction of SM-COMP*COG-COMP (*F*[1,73] = 13.81, *p* < 0.001, *η*_*p*_^2^ = 0.186). As indicated by the main effect of MODALITY, manual reactions were faster for tactile than for auditory stimuli, irrespective of sensorimotor or cognitive stimulus-response compatibility. The significant main effect of COG-COMP and the interaction of SM-COMP*COG-COMP indicated faster reactions for cognitively compatible vs. incompatible stimulus-response pairings; however, the effect was drastically reduced in the sensorimotor incompatible group (*t*[37] = 2.05, *p* = 0.019, *η*_*p*_^2^ = 0.134) compared to the sensorimotor compatible group (*t*[38] = 7.25, *p* < 0.001, *η*_*p*_^2^ = 0.527), see Fig. 2.

**Figure 3:**
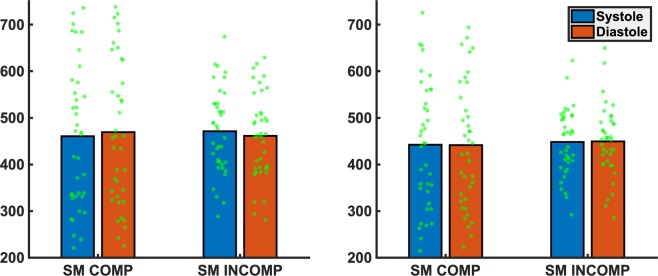
Mean reaction times and individual data points for systolic (blue) and diastolic (red) trials as a function of sensorimotor (SM Comp vs. SM Incomp) compatibility for auditory (left panel) and tactile (right panel) stimuli.

No main effect of PHASE (*F* < 1) emerged and neither did the interactions of PHASE*COG-COMP (*F*[1,73] = 2.14, *p* = 0.147, *η*_*p*_^2^ = 0.029), PHASE*COG-COMP*SM-COMP (*F*[1,73] = 3.16, *p* = 0.080, *η*_*p*_^2^ = 0.040) and PHASE*COG-COMP*SM-COMP*MODALITY (*F* < 1) reach significance. However, there was a significant interaction of PHASE*SM-COMP (*F*[1,73] = 5.02, *p* = 0.028, *η*_*p*_^2^ = 0.064) which was modulated by stimulus modality, as indicated by a significant three-way interaction of PHASE*SM-COMP*MODALITY (*F*[1,73] = 6.84, *p* = 0.012, *η*_*p*_^2^ = 0.083). To follow-up these interactions separate PHASE*SM-COMP*COG-COMP ANOVAs were run within each level of MODALITY. No significant main effect or interactions comprising the factor PHASE were evident for tactile stimuli (all *F-*values < 1), indicating the absence of cardiac-cycle-time effects in this modality. Thus, the significant interaction of PHASE*SM-COMP in the omnibus ANOVA was completely carried by auditory stimuli (*F*[1,73] = 11.52, *p* = 0.001, *η*_*p*_^2^ = 0.136). Here, a pattern emerged in which reactions were faster for systolic vs. diastolic stimulation in the sensorimotor compatible condition (*t*[37] = 2.24, *p* = 0.028, *η*_*p*_^2^ = 0.037), by contrast, reactions were slowed for systolic vs. diastolic stimulation in the sensorimotor incompatible condition (*t*[38] = 2.57, *p* = 0.012, *η*_*p*_^2^ = 0.141), see Fig. 3. Cognitive compatibility did not significantly interact with PHASE (*F*[1,73] = 2.39, *p* = 0.126, *η*_*p*_^2^ = 0.032), nor did the three-way interaction of PHASE*SM-COMP*COG-COMP reach significance (*F*[1,73] = 1.88, *p* = 0.176, *η*_*p*_^2^ = 0.025), indicating that the observed cardiac-cycle-time effects within the auditory modality were solely dependent on sensorimotor compatibility and not modulated by cognitive compatibility. The percentage of errors and responses misses was very low (combined: 4%-11%) and is given in Table 1. There were more incorrect trials in the cognitive incompatible than compatible (*F*[1,73] = 7.28, *p* = 0.009, *η*_*p*_^2^ = 0.091) and sensorimotor compatible than incompatible conditions (*F*[1,73] = 4.21, *p* = 0.050, *η*_*p*_^2^ = 0.055) but no other main or interaction effects (statistics for *F* > 1: PHASE*COG-COMP: *F*[1,73] = 1.63, *p* = 0.205, *η*_*p*_^2^ = 0.022; MODALITY*SM-COMP: *F*[1,73] = 2.54, *p* = 0.115 *η*_*p*_^2^ = 0.034; MODALITY*COG-COMP: *F*[1,73] = 2.01, *p* = 0.161 *η*_*p*_^2^ = 0.027).

**Figure 4:**
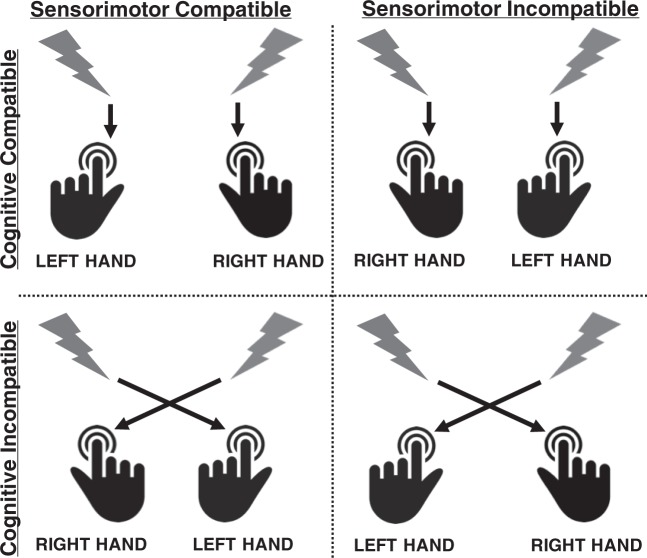
Schematic depiction of stimulus-response mappings in the sensorimotor compatible (left column), sensorimotor incompatible (right column), cognitive compatible (upper row) and cognitive incompatible (lower row) conditions.

**Table 1:** Mean values and standard deviations for sample characteristics, percentage of artifacted and incorrect trials across the four experimental groups.

	SM Comp Cog Comp	SM Comp Cog Incomp	SM Incomp Cog Comp	SM Incomp Cog Incomp
N (N female)	19 (11)	20 (12)	19 (11)	19 (11)
Age	23.7 (2.7)	23.8 (3.1)	23.5 (2.9)	23.4 (2.9)
Heart rate	77.7 (9.3)	73.1 (8.6)	76.4 (7.9)	78.2 (8.1)
Percent artifact	7.2 (5.4)	5.2 (2.5)	4.8 (3.0)	4.7 (2.8)
Percent incorrect	8.1 (1.9)	11.9 (1.9)	4.0 (1.8)	9.6 (1.9)

## Discussion

We assessed the influence of natural fluctuations in BR activity during the cardiac cycle on SRC effects at the sensorimotor and cognitive level. We found a differential influence of cardiac cycle phase on reaction times that depended solely on sensorimotor compatibility. When responses were to be made with the hand ipsilateral to stimulus presentation they were speeded in the systolic compared to the diastolic phase. The opposite pattern could be observed for sensorimotor incompatible reactions, which were slowed in the systole compared to the diastole. These effects were modality specific, only present for auditory stimuli and independent of cognitive compatibility (i.e., whether participants were instructed to react with ipsi- vs. contralateral button presses). Moreover, although startle responses were strongly modulated by cardiac cycle phase, no such sensorimotor compatibility or modality-dependent effects could be observed.

Our results challenge the notion of an overall inhibitory effect of BR activation on voluntary behavioral responses. By contrast, we found that only sensorimotor incompatible responses were inhibited, while sensorimotor compatible ones were facilitated. Within spatial SRC, sensorimotor compatibility effects are ascribed to direct automatic motor activation when stimulus and response are processed in the same hemisphere of the brain as evidenced by lateralized readiness potentials^56,57^. If the task demands sensorimotor compatible reactions, this automatic response activation facilitates quick reactions, whereas in the incompatible case it leads to interference that needs to be overridden, causing prolonged reaction times. BR activation seems to somehow potentiate this effect, and a potential underlying mechanism may be a strengthening of intrahemispheric processes and/or specific inhibition of transhemispheric traffic. However, this explanation is conflicting with our finding that BR effects on sensorimotor compatibility are restricted to auditory stimuli and absent in the tactile modality. A main difference between tactile and auditory processing is that while the somatosensory system is organized strictly contralateral, auditory signals are not completely relayed to the contralateral hemisphere^66,67^. This is also evident in our startle data showing significantly stronger laterality effects with tactile compared to auditory stimuli. At the cortical level, this results in a residual activation of the ipsilateral hemisphere which should weaken sensorimotor compatibility effects. Indeed, an absence of sensorimotor compatibility in the auditory, but not the visual domain has been reported for lateralized EEG potentials as well as reaction time distributions in a Simon task^57^. Moreover, auditory evoked potentials have been shown to be diminished in systole compared to diastole^33^. In our study, such an inhibitory BR effect could have led to a suppression of residual sensory activation in the ipsilateral hemisphere below a critical threshold necessary for automatic response activation while contralaterally, above threshold activation was preserved due to larger signal strength. As a consequence, interference due to residual activation may have been suppressed thereby facilitating sensorimotor compatible reactions. At the same time, the beneficial effect that such ipsilateral coactivation would have for sensorimotor incompatible reactions may have been abolished leading to prolongation of reaction times in this condition. While in the absence of EEG measures this explanation remains tentative, it is based on known inhibitory BR effects on sensory phenomena and fully accounts for the pattern of results obtained in this study.

Importantly, the concurrent assessment of bilateral startle responses allowed us to further disentangle the impact of BR stimulation on subcortical and cortical levels. Replicating earlier findings, we found a pronounced inhibition of startle response magnitude after systolic vs. diastolic stimulation^23–25,41^, confirming the effectiveness of the cardiac-cycle-time manipulation. To the best of our knowledge this is the first study to show that this effect extends to tactile startle stimuli as well. Of crucial importance, the pattern of results on startle responses differs markedly from the observed cardiac-cycle-time effects on manual reaction times, which were modality specific and crucially dependent on sensorimotor compatibility, that is, reversed between ipsi- vs. contralateral reactions. Therefore, it can be concluded that the behavioral effects in our study result from a specific influence of BR activation on cortical processes.

We did not find a direct influence of cardiac-cycle-time on cognitive compatibility. This result may appear surprising as recently it has been reported that systolic stimulation enhances the expression of stereotypes^46^. In this study, faces of black and white people were presented as primes during either systole or diastole and participants had to discriminate between pictures of weapons and tools presented shortly after. It was found that when black face primes were presented during systole participants were more likely to mistake tools for weapons than when primes were presented during diastole. As cognitive SRC effects have been attributed to automatic activation of a stereotype binding lateral stimuli to spatially congruent responses^68,69^, one would expect a similar effect, that is, a stronger facilitation of compatible reactions during systole compared to diastole. Nevertheless, it should be noted that the effect in the study of Azevedo *et al*. vanished when participants had to discriminate between fruits and sports objects thus an emotionally neutral component of the stereotype associating black people with sports. Here, we used startling stimuli which may also be considered threat signals, however, the available response options (left/right) might have been emotionally neutral as in the fruits/sports objects discrimination task. On the other hand, tactile startle probes are perceived as less aversive than auditory probes^70^ and since cardiac modulation of compatibility effects was restricted to auditory stimuli, this might also reflect an effect dependent on the affective quality of the stimuli. Therefore, our results are in line with the previous findings suggesting that BR enhanced automatic activation of response tendencies is dependent on the emotional nature of the stimuli.

Although BR modulatory effects were restricted to sensorimotor compatibility, our results should not be interpreted as being “uncognitive”, as sensorimotor and cognitive processes are deeply interwoven at the neural level, i.e. motor cognition^71^. In fact, the three-way interaction effect indicating a moderation of the observed cardiac-cycle-time effects by cognitive compatibility just barely missed significance. However, descriptively, suppressive BR effects in the sensorimotor incompatible condition were strongest with a cognitive compatible mapping and attenuated in the cognitive incompatible condition, reminiscent of hierarchical interaction models between sensorimotor and cognitive compatibility effects previously proposed^72,73^. Furthermore, the magnitude of the cardiac-cycle-time effect in our data reached a maximum of 18 ms while the interhemispheric transmission time as measured through unimanual Poffenberger tasks is in the order of two to four ms^61,74^. Thus, it appears likely that the suppression of residual noise described above affected the cognitive processes associated with sensorimotor compatibility, which in this specific case led to a strengthening of both their interfering and facilitating consequences. Albeit appearing rather specific, such a suppression of sensory noise below a critical threshold might represent a general mechanism underlying previously reported cognitive cardiac-cycle-time effects. For instance, in a study using visual stimuli Pramme *et al*.^43^ showed that BR activation during the cardiac cycle reduced the impact of a masking stimulus presented shortly before the target leading to improved detection performance during systolic stimulation. Similarly, in a visual selection task the same authors also found that the influence selection difficulty (based on the type of distractors) had on the ability to select the target was attenuated for stimuli presented during systole compared to diastole^44^. That is, rather than directly affecting cognitive operations, BR activity may change the cortical representation these cognitive processes act upon in terms of the signal to noise ratio and thereby reduce interference in conflict tasks.

Given that rises in blood pressure are a typical characteristic of the stress response e.g^75–77^ after the detection of a threatful situation the assumption of an overall inhibitory effect of BR activation seems paradoxical. From a survival perspective, one would rather expect a facilitative influence enabling quick reactions to escape a dangerous situation. In the same vein it seems counterintuitive that an important protective reflex such as the startle response is inhibited by BR stimulation. Our results offer an explanation for this apparent paradox, suggesting that the suppression of subcortical reflex circuits goes along with a facilitation of a certain kind of voluntary cortically mediated responses. Interestingly, this facilitation is limited to spatially compatible reactions which represent the most appropriate response to a lateralized threat signal. Bluntly put, when you hear something explode to your right the quickest way to shield yourself from a pending impact would be a right-sided movement. BR activation seems to facilitate just that while inhibiting the probably inappropriate contralateral reaction. Seen this way, heightened BR activation during stress and ensuing inhibitory effects may very well be adaptive in danger situations.

We synchronized stimulus presentation to the ECG in order to target natural fluctuations in the firing rate of arterial baroreceptors during systole vs. diastole. As described for instance by Edwards *et al*.^78^ BR activity is increased during systole in a time interval ranging from approximately 90–340 ms after the R-wave, with maximal activation occurring around 250 ms. We presented stimuli at R + 230 ms and R + 530 ms, since previous research indicates reliable startle modulation at these intervals^25,41^. These timings are generally accurate with a resting heart rate, but accuracy in targeting systole and diastole will deteriorate with stress level heart rates (i.e. >120 bpm) which lead to a substantial shortening of the cardiac cycle, disproportionally affecting the diastolic phase. However, such high heart rates were not observed in the current study nor did experimental groups differ in heart rate. Moreover, replicating previous findings, we observed robust startle inhibition during systole compared to diastole across groups, which also indicates the validity of the chosen timings. Cardiac-cyle-time effects have been shown to be causally dependent on intact visceroafferent signal transmission, as they are absent or strongly attenuated in diabetic neuropathy^25,79^. Nevertheless, besides variations in BR activity, other concomitant changes during the cardiac cycle have recently been shown to impact on central-nervous activity (i.e. “vasculo-neural coupling”)^80^ and may have also influenced the observed results.

Some further limitations of the current study need to be considered. Since we used startling stimuli as targets in this experiment, it is questionable in how far our results maybe be specific to startle. Startle stimuli are highly salient threat signals and previous research has shown a specific cardiac-cycle-time effects on emotional processing, threat in particular^46,50,51,81^. Thus, there is reason to assume that differential BR effects surface within emotional contexts and further research is needed to determine whether our findings are to be generalized beyond startle. Moreover, we did not employ visual stimuli, and as we found modality- specific effects, it will be interesting to see if different results emerge in the visual domain. Finally, the relative contributions of sensorimotor and cognitive processes mediating SRC effects are still a matter of debate and additive, interactive as well as hierarchical influences have been proposed^55,56,73^. A vast array of operational variations have been employed to reveal such specific contributions (see e.g.^82^ ^for review^) and it can be expected that these will also modulate the influence of cardiac cycle time.

In conclusion, the assumption of a general BR mediated behavioral inhibition appears to be overly simplified. It may rather be assumed that inhibitory effects at the cortical level differentially affect sensorimotor and cognitive processes to induce complex changes in information processing which may facilitate certain behavioral responses while inhibiting others. This study exemplifies how such differential effects may play out to influence performance in conflict tasks and further shows that SRC paradigms may offer a viable method to uncover the complex interactions underlying behavioral BR effects.

## Methods

### Sample

The sample consisted of 80 healthy men and women (mean age: 23 years, SD: 2.9 years) recruited via email digest at the University of Trier. As in previous publications, e.g.^77^, participation in the study was limited to right-handed, healthy people with normal weight (Body Mass Index between 19 and 25) and age between 18 and 35 years. Applicants were not included if they showed any evidence of acute or chronic diseases of the cardiovascular system (deviations from sine rhythm, glaucoma, Raynaud’s disease, history of fainting, resting blood pressure above 140/90 mmHg), history of psychiatric disease or family history of arterial hypertension. Further exclusion criteria were smoking of more than five cigarettes per day, drug intake or current use of medication. A personal screening interview determined if all criteria for inclusion in the study were met. All participants gave written informed consent. They were compensated with 15 € after completion of the experiment. Two participants needed to be excluded due to excessive artifact contamination (see 2.4) and another one due to loosening of ECG electrodes during the experiment, reducing the final sample size to *N* = 77. Sample characteristics are given in Table 1.

### Procedure and experimental task

Participants were sitting comfortably in an armchair in front of an LCD computer screen, with a viewing distance of 80 cm. A white fixation cross was continuously displayed in the middle of the screen throughout testing and participants were asked to look at the fixation cross during the experimental blocks. After electrodes for ECG and EMG measurement had been placed, participants were told that in the upcoming experiment they would be presented with air-puffs and noise bursts and that they should react to them according to the instructions presented on screen using the response box in front of them. Onscreen instructions differed between participants depending on the experimental condition they had been assigned to. There were four experimental conditions orthogonally varying cognitive and sensorimotor stimulus-response-compatibility: with respect to stimulus laterality, responses had to be made with either the ipsi- or contralateral hand (sensorimotor compatibility) on either the ipsi- or contralateral response button (cognitive compatibility). Onscreen instructions first informed participants to either react to right stimuli with right and to left stimuli with left button presses (cognitively compatible condition) or vice versa (cognitively incompatible condition). Participants were then instructed to either place their right and left index fingers on the right and left buttons, respectively, or vice versa (left finger/right button, right finger/left button) so that irrespective of cognitive compatibility responses to left and right stimuli could be made with left and right index fingers, respectively (sensorimotor compatible condition), or vice versa (sensorimotor incompatible condition). See Fig. 4 for a graphical depiction of the design.

The experiment then commenced with twelve practice trials in which six air-puffs and six noise bursts were presented in alternating order. After that, the main part of the experiment started consisting of 160 trials in total (20 per condition) presented with a jittered intertrial interval of five to eight seconds and organized in two equally sized blocks separated by a two minutes break. Trial condition varied randomly with respect to stimulus modality (noise bursts vs. airpuffs), laterality (left vs. right ear/temple) and cardiac cycle phase (230 ms vs 530 ms after peak of R-wave). After completion of the experiment, electrodes were removed and participants were thanked, compensated and dismissed. All procedures were approved by the ethics committee of the states medical association (Landesärztekammer Rheinland-Pfalz) and in accordance with the Declaration of Helsinki. All participants provided written informed consent and their rights were protected.

### Stimulus presentation

Unilateral auditory and tactile startle probes were used as stimuli. Auditory stimuli were short white noise bursts (105 dB(A), instantaneous rise time, duration 50 ms) presented monaurally via headphones (Holmco PD-81, Holmberg GmbH & Co. KG). Air-puffs delivered with a pressure of 10 psi via tubes mounted on the headphones and directed to the right and left temples were used as tactile startle probes. All stimuli were presented either 230 ms (systole) or 530 ms (diastole) after the peak of the R-wave in the ECG^24,25,41^. ECG electrodes were placed in lead II configuration and online detection of R-waves was performed by an AccuSync 72 ECG monitor (AccuSync Medical Research Corporation) delivering TTL pulses. Timing of stimulus presentation was then controlled by E-Prime 2.0 (PST Software, Inc) running on a Windows PC connected to the ECG monitor via serial interface.

### Startle EMG recording and analysis

Startle EMG was measured and scored following previously described standards, e.g.^25,83^. Startle responses were assessed bilaterally via orbicularis oculi EMG using two Ag/AgCl electrodes (24 mm diameter) placed below the left and right eye with an interelectrode distance of 1.5 cm. The reference electrode was taped to the forehead. EMG was recorded with DASYLab software at a sampling rate of 1000 Hz (50 Hz notch filter; bandpass filter 30–500 Hz). Data were rectified and integrated with a time constant of 10 ms. A customized C++ based semi-automated PC program was used to analyze EMG responses. The algorithm identified response peaks in the rectified and integrated signal during a time interval of 20 to 150 ms after the startle probe onset. The baseline period was defined as 50 ms window preceding stimulation. Trials were visually inspected for artifacts (i.e., trials with excessive background noise, multiple peaks, coinciding blinks) offline and invalid trials discarded. For data analysis, we used only data of participants with at least 75% artifact-free trials; two participants needed to be excluded for that reason. The percentage of artifacted trials did not differ between conditions and is given in Table 1. If responses were not visible (zero amplitude) at the typical response latency of a particular participant, response amplitude was set to zero. Zero response data were included in the average yielding startle response magnitude as the final output measure. Startle data were normalized (z-scored) within-participant^65^, averaged separately for each condition, and according to whether startle was measured ipsilateral or contralateral to stimulus presentation (analog to sensorimotor compatibility for manual responses). Before within-subjects standardization, we assessed whether startle responding differed between experimental groups in the raw data, this was not the case (all *F*-values < = 1), see Supplementary Fig. S1 for a depiction of the raw data.

### Statistical analyses

Separate mixed-model analyses of variance (ANOVAs) were conducted on reaction time and startle data. Startle data was analyzed in a MODALITY (tactile vs. auditory) * PHASE (systole vs. diastole) * SIDE (ipsi- vs. contralateral) repeated-measures ANOVA. Manual reaction times were subjected to a mixed-model ANOVA comprising the within-subject factors MODALITY and PHASE as well as the between-subject factors SM-COMP (sensorimotor compatible vs. incompatible) and COG-COMP (cognitive compatible vs. incompatible). Results with an alpha error probability below 5% were deemed significant. Partial eta squared is reported as a measure of effect size. Significant interactions were followed up by ANOVAs and *t*-tests as appropriate.

## Supplementary information

Supplementary information

## Data Availability

The datasets generated during and/or analysed during the current study are available from the corresponding author on reasonable request.
