# Supplementary material for: Disentangling sensorimotor and cognitive cardioafferent effects: A cardiac-cycle-time study on spatial stimulus-response compatibility
Source: Sci Rep. 2020 Mar 4;10:4059. doi: 10.1038/s41598-020-61068-1 (PMC7055319; doi:10.1038/s41598-020-61068-1)
Supplement: Supplementary file 1 — Supplementary information [file 41598_2020_61068_MOESM1_ESM.docx]

**Supplementary material:**

**Disentangling sensorimotor and cognitive cardioafferent effects: A cardiac-cycle-time study on spatial stimulus-response compatibility**

Mauro F. Larra^1^*, Johannes Finke^2^, Edmund Wascher^1^ & Hartmut Schächinger^2^

**^1^** Leibniz Research Centre for Working Environment and Human Factors, 44139 Dortmund, Germany

**^2^** Division of Clinical Psychophysiology, Institute of Psychobiology, University of Trier, 54290 Trier, Germany


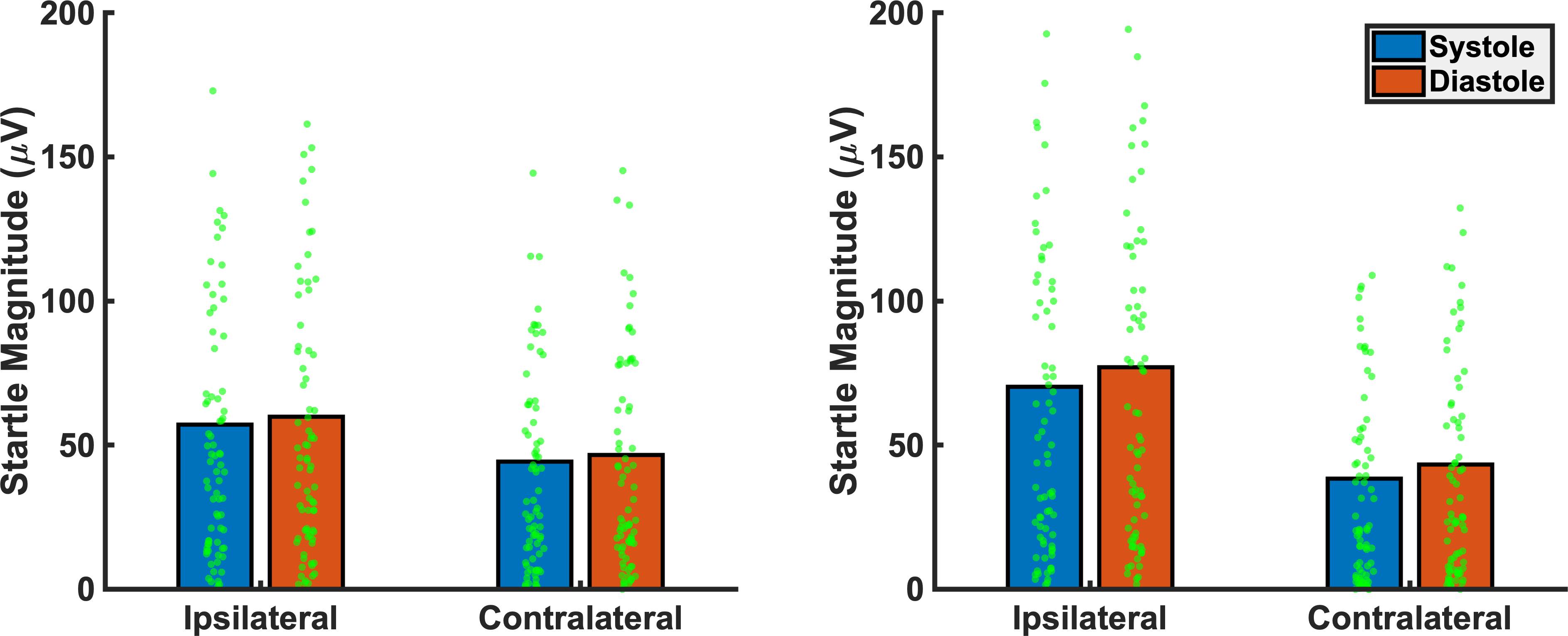


Supplementary Fig.S1: Startle magnitude (raw data) in systolic (blue) and diastolic (red) trials measured at the eye ipsilateral vs. contralateral to stimulus presentation for auditory (left panel) and tactile (right panel) stimuli.
